# Supplementary material for: Flutamide Promotes Early Hepatocarcinogenesis Through Mitophagy in High-Fat Diet-Fed Non-Obese Steatotic Rats
Source: Int J Mol Sci. 2025 Mar 17;26(6):2709. doi: 10.3390/ijms26062709 (PMC11943065; doi:10.3390/ijms26062709)
Supplement: Supplementary file 1 [file ijms-26-02709-s001.zip › ijms-3414758-supplementary.pdf]

Supplemental Materials

Flutamide promotes early hepatocarcinogenesis in high-fat diet-fed non-obese steatotic rats through mitophagy

Emika Hara[1], Kanami Ohshima[1], Mio Takimoto [1], Yidan Bai [1], Mai Hirata [1], Wen Zeng[1], Suzuka Uomoto[1], Mai Todoroki [1,2], Mio Kobayashi[1,2], Takuma Kozono [3], Tetsuhito Kigata[4], Makoto Shibutani[1], Toshinori Yoshida[1*]

[1] Laboratory of Veterinary Pathology, Cooperative Department of Veterinary Medicine, Tokyo University of Agriculture and Technology, 3-5-8 Saiwai-cho, Fuchu-shi, Tokyo 183-8509, Japan

[2] Cooperative Division of Veterinary Sciences, Tokyo University of Agriculture and Technology, 3-5-8 Saiwai-cho, Fuchu-shi, Tokyo 183-8509, Japan

[3] Smart-Core-Facility Promotion Organization, 3-5-8 Saiwai-cho, Fuchu-shi, Tokyo 183-8509, Japan

[4] Laboratory of Veterinary Anatomy, Cooperative Department of Veterinary Medicine, Tokyo University of Agriculture and Technology, 3-5-8 Saiwai-cho, Fuchu-shi, Tokyo 183-8509, Japan

*Corresponding author: Toshinori Yoshida, D.V.M., Ph.D., Laboratory of Veterinary Pathology, Tokyo University of Agriculture and Technology, 3-5-8 Saiwai-cho, Fuchu-shi, Tokyo 183-8509, Japan.
E-mail: yoshida7@cc.tuat.ac.jp; Tel +81-42-367-5874; Fax: +81-42-367-5771

Supplemental Materials and Methods

Real-Time Reverse Transcription-Polymerase Chain Reaction Analysis

Expression analysis at the mRNA level was performed using the primers listed in Table S6, as previously reported [28]. Total RNA was extracted from liver samples from 6 animals each in the BD, BD+FL, HFD, and HFD+FL groups using RNeasy Mini Kits (Qiagen, Hilden, Germany). Single-stranded cDNA was extracted from 2 μg of RNA using dithiothreitol, deoxynucleoside triphosphates, random primers, RNaseOUT and SuperScriptTM III Semiquantitative real-time RT-PCR was performed with Power SYBR® Green PCR Master Mix (Life Technologies, Carlsbad, CA, U.S.A.). Semi-quantitative real-time RT-PCR was performed using the Applied Biosystems StepOnePlusTM Real-Time PCR System (Life Technologies). Primers for each gene were designed using Primer Express 3.0 software (Life Technologies). The mRNA expression levels of each gene were calculated by the $2^{-\Delta\Delta C_T}$ method, corrected for the threshold cycle (CT) value of the hypoxanthine phosphoribosyl transferase (Hprt1) gene as an endogenous control.

Table S1. Body weight change intake in rats

Group	BD	BD+FL	HFD	HFD+FL
No. of animals	6	6	6	6
1‡	165.0±4.5	165.2±4.0	164.1±5.2	159.7±5.4
2	151.8±5.4	158.7±7.3	151.5±6.1	152.6±10.8
3	191.1±7.5	194.0±6.1	190.6±6.0	191.0±7.9
4	221.4±9.8	219.7±4.3	216.3±8.0	214.9±7.1
5	217.9±11.2	210.6±7.4	214.2±10.2	204.5±9.4
6	238.1±10.8[a]	227.8±5.7[ab]	234.6±9.4[ab]	222.3±8.2[b]
7	252.1±11.0[a]	236.0±5.6[b]	246.8±12.1[ab]	231.5±9.5[b]
8	266.7±12.8[a]	248.6±4.5[b]	261.4±12.1[ab]	245.0±9.3[b]
9	269.9±13.5[a]	254.3±4.3[ab]	266.7±12.2[ab]	250.1±9.3[b]
10	277.7±13.9[a]	257.5±5.3[bc]	272.1±11.0[ab]	254.7±10.8[c]
11	283.6±14.4[a]	264.5±4.8[b]	281.1±12.2[ab]	265.5±12.6[ab]
12	292.9±13.2[a]	269.7±5.3[c]	289.0±12.4[ab]	271.3±13.2[bc]
13	298.5±14.6[a]	269.9±6.5[b]	290.5±14.1[a]	268.2±10.2[b]

Abbreviations: BD, basal diet; HFD, high-fat diet; FL, flutamide.

‡: Study week.

Data are shown as the mean ± standard deviation(g).

Different letters indicate significant differences between groups ($p<0.05$, significantly different byTukey's or Steel-Dwass test).

Table S2. Food intake in rats

Group	BD	BD+FL	HFD	HFD+FL
No. of cages	2	2	2	2
1‡	19.6	24.2	7.1	7.5
2	86.9	84.9	68.8	68.6
3	68.4	63.7	54.7	46.4
4	33.2	29.4	22.6	20.8
5	58.5	56.3	40.1	39.4
6	51.2	50.1	39.3	38.0
7	49.9	46.5	36.5	33.6
8	47.4	46.7	32.8	30.9
9	42.0	42.7	29.8	31.1
10	40.8	41.5	30.4	32.6
11	41.9	38.6	31.0	31.7
12	38.5	37.5	26.0	27.1
13	34.3	35.3	23.9	25.6

Abbreviations: BD, basal diet; HFD, high-fat diet; FL, flutamide.

‡: Study week.

Data are shown as the mean (g/kg).

Table S3. Water intake in rats

Group	BD	BD+FL	HFD	HFD+FL
No. of cages	2	2	2	2
1‡	73.3	71.5	69.8	62.2
2	118.7	112	99.3	95.8
3	88.2	82.7	70.1	67.0
4	71.3	64.8	58.6	51.0
5	73.9	70.0	57.7	56.6
6	65.3	62.2	51.8	48.9
7	62.2	60.8	48.2	46.8
8	58.5	59.3	43.8	45.6
9	54.1	52.0	42.4	41.3
10	52.8	51.8	41.5	41.3
11	50.6	48.1	40.2	38.0
12	48.2	50.0	37.8	36.5
13	48.9	52.5	38.5	36.5

Abbreviations: BD, basal diet; HFD, high-fat diet; FL, flutamide.

‡: Study week.

Data are shown as the mean (g/kg).

Table S4. Immunohistochemistry of Ki-67 in the preneoplastic liver foci of rats

Group	BD	BD+FL	HFD	HFD+FL
No. of animals	6	6	6	6
Ki-67 labeling index (%)	5.4±1.5	7.3±2.3	5.2±2.3	7.5±3.5

Abbreviations: BD, basal diet; HFD, high-fat diet; FL, flutamide.

Data are shown as the mean ± standard deviation.

Different letters indicate significant differences between groups ($p<0.05$, significantly different by Tukey's or Steel-Dwass test).

Table S5. Antibodies for immunohistochemistry

Antigen	Host species	Clonality (Clone)	Dilution	Antigen retrieval	Manufacture
AMBRA1	Rabbit	Polyclonal	1:400	MW [b]	Abcam (Cambridge, UK)
GST-P	Rabbit	Polyclonal	1:1000	None	Medical & Biological Laboratories (Nagoya, Japan)
Ki-67	Rabbit	Monoclonal (SP6)	1:500	AC [c]	Abcam (Cambridge, UK)
LC3B	Rabbit	Polyclonal	1:400	MW [a]	Abcam (Cambridge, UK)
Parkin/PARK2	Rabbit	Polyclonal	1:200	MW [b]	Bioss ANTIBODIES (Boston, America)
PINK1	Rabbit	Polyclonal	1:100	MW [a]	Abcam (Cambridge, UK)

Abbreviations: MW, Microwaving; AC, Autoclaving

[a] at 90°C for 10 min in citrate buffer, pH6.0

[b] at 90°C for 10 min in citrate buffer, pH9.0

[c] at 121°C for 10 min in citrate buffer, pH6.0

Table S6. Sequence of primers used for real-time RT-PCR

Accession no.	Gene	Forward primer (5`→3`)	Reverse primer (5`→3`)
NM_134394.2	*Atg3*	GCAGCACCATGCAGGTGAG	TGGTCACTCGGTCCAGGATC
NM_001014250.1	*Atg5*	CTGTTCGATCTTCTTGCATCA	TCCTTTTCTGGAAAACTCTTGAA
NM_001012097.1	*Atg7*	TTCTTAGAAGATTTGACTGGTCTTACA	TCACTCATGTCCCAGATCTCA
NM_012857	*Lamp1*	GCAAGGCGCTCGCCCTCAAT	GCCCGCGTGACTCCTCTTCC
NM_017068	*Lamp2*	AGCAGGTGGTTTCCGTGTCTCG	AGGGCTGCTCCCACCGCTAT
NM_022867	*LC3*	CGGGTTGAGGAGACACACAA	TCTTTGTTCGAAGCTCCGGC
NM_175843.4	*p62*	CGGAAGTCAGCAAACC	ATGCGTCCAGTCGTCA
XM_032903710.1	*AMBRA1*	TTTAACCAGGAGACCGGCCA	TCACCACTGTCCCTCGCTG
XM_032894705.1	*Parkin*	GGTCCAGCTAAACCCACCTAC	GAACGATGGCTCCCAAAGACA
NM_001106694.1	*PINK1*	GTGTCTGACCCACTGGACAC	CTGCTCCCTTTGAGACGACA
NM_053420.3	*BNIP3*	AAGCGCACAGCTACTCTCAG	AGTGGAAGTTGTCAGACGCC
XM_032913026.1	*NADH*	GTAGTACTGCGCTCGTTTTCG	GGTTTGGCATTGACTGGCTC
NM_198788.3	*SDHD*	ACATCCACCTGTCACCAAGC	CTTGTCCAATGCCCCAGTGA
NM_138883.1	*ATP synthase*	TATGCAACCGCCCTGTACTC	TGGGGTCCTTCAAGAGTTGC
NM_178095.3	*Abca1*	CCAGGAGCGTGTGAGCAAAG	ACCAGTGTAGCAGGGACCACATAA
NM 017340	*Acox1*	GCGCAAGGAGCGGGCCTCC	CTCGACGGCGCCGGGTATTC
NM_019287.2	*Apob*	TGCGGTGGCAGAAATAACG	AGTAGCCAGAGAGCTGGTCTGA
NM 001012345	*Dgat2*	CTTCCTGGTGCTAGGAGTGG	GCCAGCCAGGTGAAGTAGAG
NM 017332	*Fasn*	GCGGGCGTGGTAATGCT	CTGTTCGCAAATACGCTCCAT
M33648	*Hmgcs1*	CCAGGCACTTGGTACCTTGAA	GGGACGCCGGGCATA
NM 012583	*Hprt*	GTCAAGCAGTACAGCCCCAAA	CAACACTTCGAGAGGTCCTTTTC
XM_032897642	*Hsd3b1*	CCCTGCTCTACTGGCTTGC	TCTGCTTGGCTTCCTCCC
L03294	*Lpl*	ATCTGAGCCTGACCAAGAACTAAAC	AAGCCAAGGCAGGATGGTT
NM_031049	*Lss*	GACTAAGCGTGGCGGGTATTTG	GCATCACTGCGGAGGTACACTCTA
NM 013196	*Ppara*	CCCCACTTGAAGCAGATGACC	CCCTAAGTACTGGTAGTCCGC
NM 001145366	*Pparg*	GACCACTCCCATTCCTTTGA	CATTGGGTCAGCTTGTGA

NM 139192	*Scd1*	CACACGCCGACCCTCACAACT	TCCGCCCTTCTCTTTGACAGCC
NM_001276707.1	*Srebf1*	GGAGCCATGGATTGCACATT	GCTTCCAGAGAGGAGCCCAG
NM 001033694	*Srebf2*	TTCAGCACCGCTCCACAGA	GCACCTGCTGCTGGATGGTA
NM_012540.3	*Cyp1a1*	GAAGAAGCTAATCAAAGAGCAC TACAGG	CAATGCTCAATGAGGCTGTCTG
NM_001134844.1	*Cyp2b1*	GGCTCACACCGGCTACCAA	TGAAAACCTCTGAATCTCGT GGATA
NM_013105.2	*Cyp3a1*	GTAAAATACTTGAGGCAAGAGA AAGGC	TCGGGTTGTTGAGGGAATCA
NM_012675.3	*Tnf-a*	ATACACTGGCCCGAGGCAAC	CCACATCTCGGATCATGCTTTC
NM 012520	*Catalase*	ATTGCCGTCCGATTCTCC	CCAGTTACCATCTTCAGTGTAG
NM 030826	*Gpx1*	GCTGCTCATTGAGAATGTCG	GAATCTCTTCATTCTTGCCATT
NM_183403.2	*Gpx2*	GTGTGATGTCAATGGGCAGAAT	AGGGCAGCTTGTCTTTCAGGTA
NM 17051	*Mn-SOD*	GACCTGCCTTACGACTATG	TACTTCTCCTCGGTGACG
NM_017050.1	*Sod1*	CATTCCATCATTGGCCGTACTA	TTTCCACCTTTGCCCAAGTC
NM_017051.2	*Sod2*	CTCCCTGACCTGCCTTACGA	CTGCATGATCTGCGCGTTA

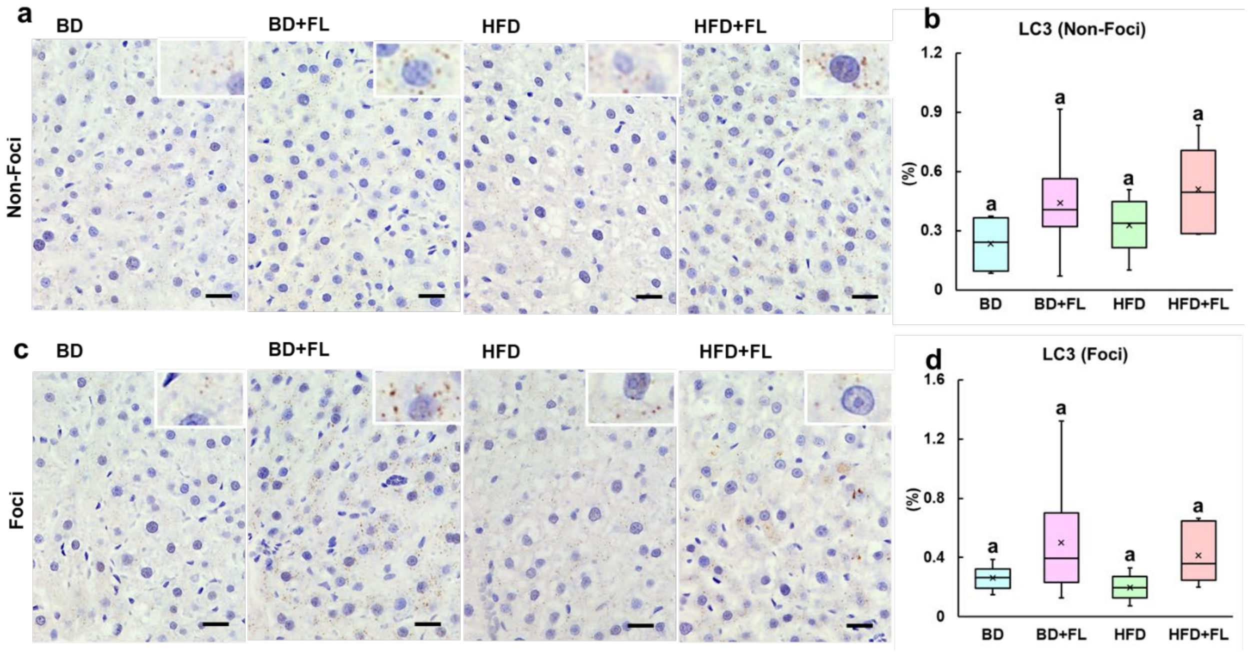

Figure S1. Representative images and quantitative analysis of LC3-positive granules in background hepatocytes and preneoplastic lesions. (a, b) Representative images and box plots illustrating LC3-positive granules in background hepatocytes (non-foci). (c,d) Representative images and box plots depicting LC3-positive granules in preneoplastic lesions. Different letters indicate significant intergroup differences ($p < 0.05$, Tukey's test or Steel–Dwass test).

Abbreviations: BD = basal diet; FL = flutamide; HFD = high-fat diet; LC3, LC3 = microtubule-associated protein 1A/1B-light chain 3.

Individual data pf Figure S1b.

The ratio (%) of LC3 expression in non-foci (background hepatocytes)..

Group:	BD	BD+FL	HFD	HFD+FL
	0.24	0.41	0.42	0.28
	0.37	0.92	0.51	0.29
	0.36	0.40	0.10	0.83
	0.25	0.07	0.25	0.52
	0.10	0.45	0.26	0.66
	0.08	0.40	0.43	0.47
Mean	0.23	0.44	0.33	0.51
SD	0.12	0.27	0.15	0.22

Individual data of Figure S1d

The ratio (%) of LC3 expression in foci (neoplastic lesions).

Group:	BD	BD+FL	HFD	HFD+FL
	0.26	0.41	0.33	0.20
	0.39	1.32	0.25	0.32
	0.30	0.38	0.15	0.66
	0.20	0.13	0.07	0.40
	0.27	0.50	0.21	0.64
	0.15	0.27	0.19	0.26
Mean:	0.26	0.50	0.20	0.41
SD:	0.08	0.42	0.09	0.20

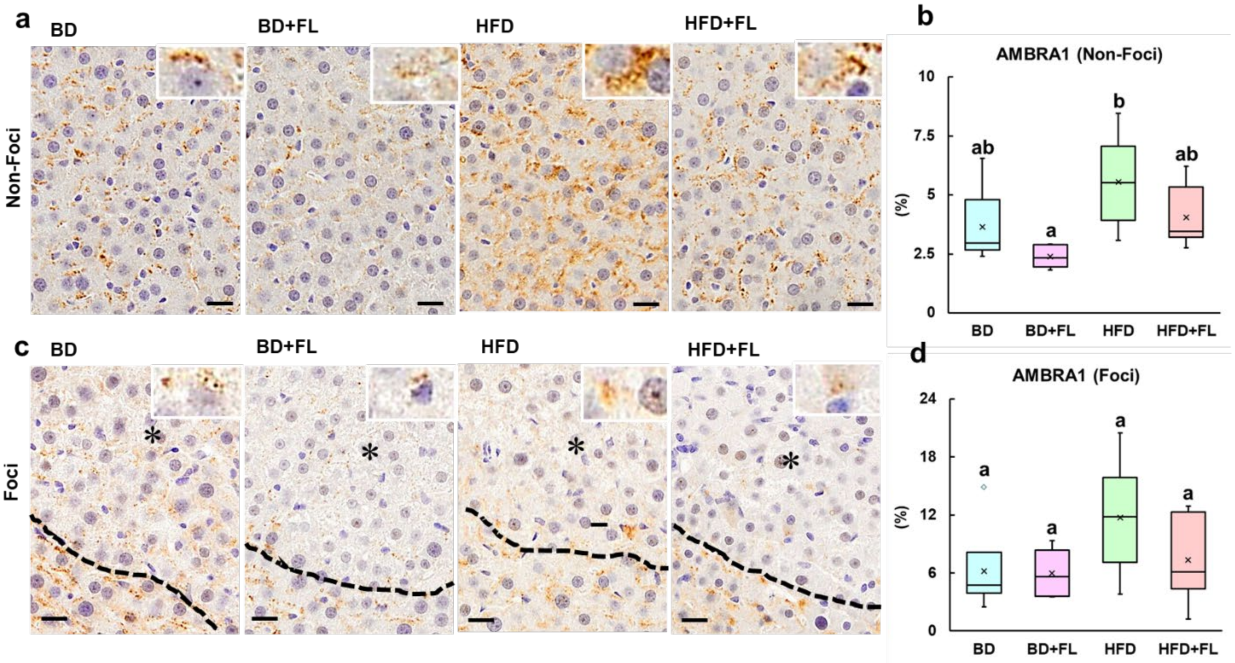

Figure S2. Representative images and quantitative analysis of AMBRA1-positive granules in background hepatocytes and preneoplastic lesions. (a, b) Representative images and box plots illustrating AMBRA1-positive granules in background hepatocytes (non-foci). (c, d) Representative images and box plots depicting AMBRA1-positive granules in preneoplastic lesions (* = foci). Different letters indicate significant intergroup differences ($p < 0.05$, Tukey's test or Steel-Dwass test). Abbreviations: AMBRA1 = autophagy and Beclin 1 regulator 1; BD = basal diet; * = foci; FL = flutamide; GST-P = placental glutathione S-transferase; HFD = high-fat diet.

Individual data of Figure S2b.

The ratio (%) of AMBRA1 expression in non-foci (background hepatocytes).

Group:	BD	BD+FL	HFD	HFD+FL
	4.2	2.9	4.2	3.5
	2.7	2.0	4.9	5.0
	6.5	2.9	3.1	2.8
	2.8	2.3	6.6	3.4
	2.4	2.4	6.2	3.4
	3.2	1.8	8.5	6.2
Mean:	3.6	2.4	5.6	4.0
SD:	1.6	0.5	1.9	1.3

Individual data of Figure S2d.

The ratio (%) of AMBRA1 expression in foci (neoplastic lesions).

Group:	BD	BD+FL	HFD	HFD+FL
	5.9	9.4	14.4	5.6
	5.1	8.1	13.0	12.1
	14.9	6.8	8.2	12.9
	2.5	3.6	3.8	6.6
	4.4	3.6	20.5	1.2
	4.4	4.5	10.6	5.4
Mean:	6.2	6.0	11.7	7.3
SD:	4.4	2.5	5.7	4.4

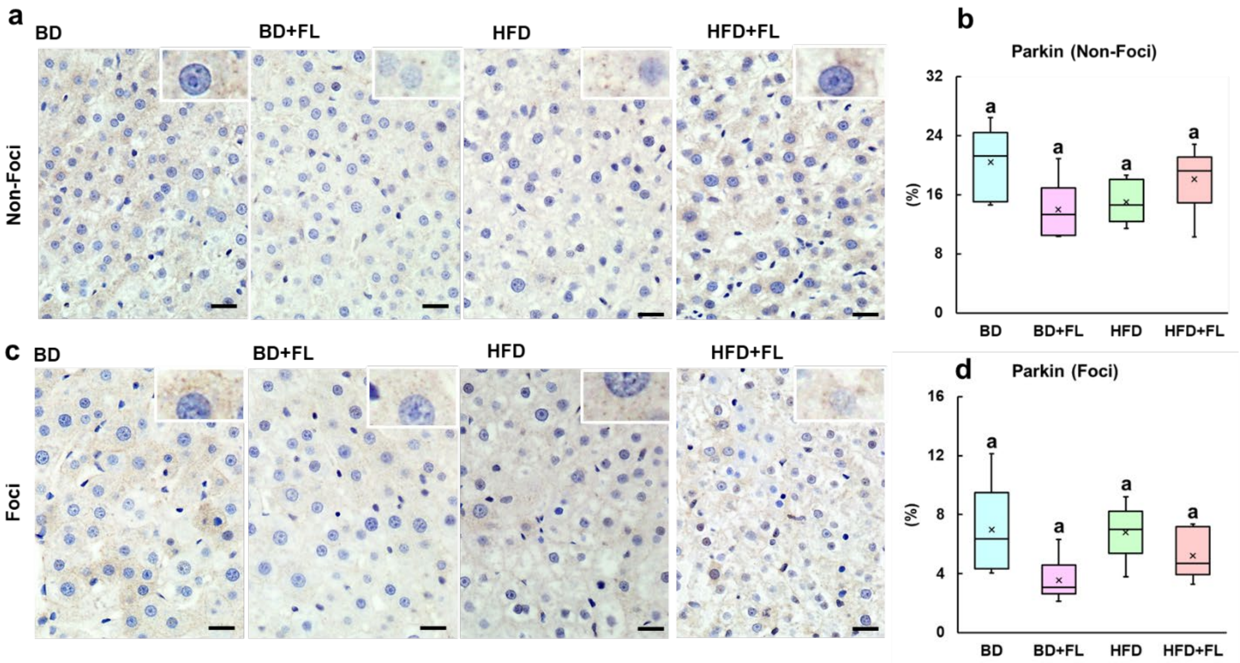

Figure S3. Representative images and quantitative analysis of Parkin-positive granules in background hepatocytes and preneoplastic lesions. (a, b) Representative images and box plots illustrating Parkin-positive granules in background hepatocytes (non-foci). (c, d) Representative images and box plots depicting Parkin-positive granules in preneoplastic lesions. Different letters indicate significant intergroup differences ($p < 0.05$, Tukey's test or Steel–Dwass test). Abbreviations: BD = basal diet; f=foci; FL = flutamide; HFD = high-fat diet.

Individual data pf Figure S3b.

The ratio (%) of PARKIN expression in non-foci (background hepatocytes).

Group:	BD	BD+FL	HFD	HFD+FL
	21.1	15.6	15.4	20.5
	21.4	20.9	17.9	16.5
	26.5	14.0	12.8	20.6
	14.7	10.6	13.9	10.3
	23.7	12.6	18.7	22.9
	15.2	10.4	11.4	17.9
Mean:	20.4	14.0	15.0	18.1
SD:	4.7	3.9	2.9	4.4

Individual data of Figure S3d.

The ratio (%) of PARKIN expression in foci (neoplastic lesions).

Group:	BD	BD+FL	HFD	HFD+FL
	8.6	2.8	9.2	4.8
	4.0	6.3	7.7	7.4
	12.1	4.0	5.9	7.1
	4.4	2.9	3.8	4.6
	5.1	3.2	7.9	4.2
	7.6	2.1	6.3	3.3
Mean:	7.0	3.6	6.8	5.2
SD:	3.1	1.5	1.9	1.7

a

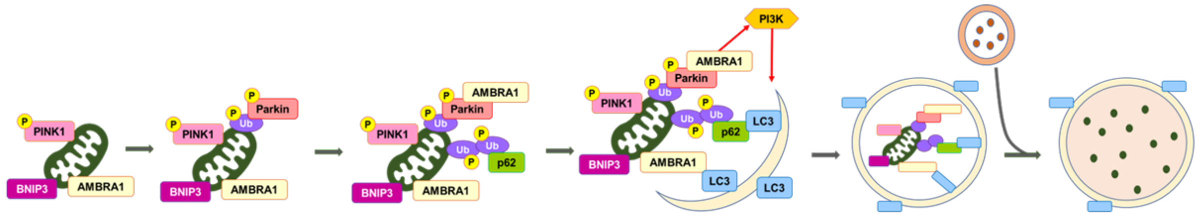

b

	Early inhibition	Late inhibition	Induction
LC3	↓	↑	↑ / ↓
AMBRA1	↑	↑	↓
Parkin	↑ (Parkin-dependent)<br>↓ (Parkin-independent)	↑ (Parkin-dependent)<br>↓ (Parkin-independent)	↓ (Parkin-dependent)<br>↓ (Parkin-independent)

Figure S4. Hypothetical expression of mitophagy markers during autophagic flux. (a) Mitochondrial priming is mediated by Parkin-dependent or -independent mechanisms. Abnormal mitochondria constitutively express non-degraded PINK1 on the outer mitochondrial membrane, leading to the phosphorylation of parkin and ubiquitin. Phosphorylated PARKIN- and ubiquitin-tagged mitochondria are sequestered by LC3, along with the mitophagy-related receptors p62 and BNIP3 to form autophagosomes, which in turn fuse with lysosomes to form autolysosomes. In Parkin-dependent mitophagy, activated Parkin interacts with AMBRA1 on the outer mitochondrial membrane, promoting mitophagy. AMBRA1 directly binds to LC3 and promotes autophagosome formation. (b) Autophagic flux consists of three stages: early inhibition, late inhibition, and induction. Early inhibition involves low LC3 expression and a high AMBRA1 expression; high Parkin levels indicate Parkin-dependent mitophagy, whereas a low Parkin level imply Parkin-independent mitophagy. Late inhibition involves high expression of both LC3 and AMBRA1, with high Parkin expression indicating Parkin-dependent pathway, and low Parkin expression indicating Parkin-independent pathway. The induction of autophagy is defined by low or high LC3 expression levels and low AMBRA1 expression, where low Parkin expression signifies Parkin-dependent or -independent pathway. (C) Immunohistochemical expression of Parkin, AMBRA1, and LC3 during mitophagy. Abbreviations: AMBRA1 = autophagy and Beclin 1 regulator 1, BNIP3 = BCL2/adenovirus E1B-interacting protein 3, LC3 = microtubule-associated protein 1A/1B-light chain 3, PINK1 = phosphatase and tensin homolog (PTEN)-induced kinase 1.

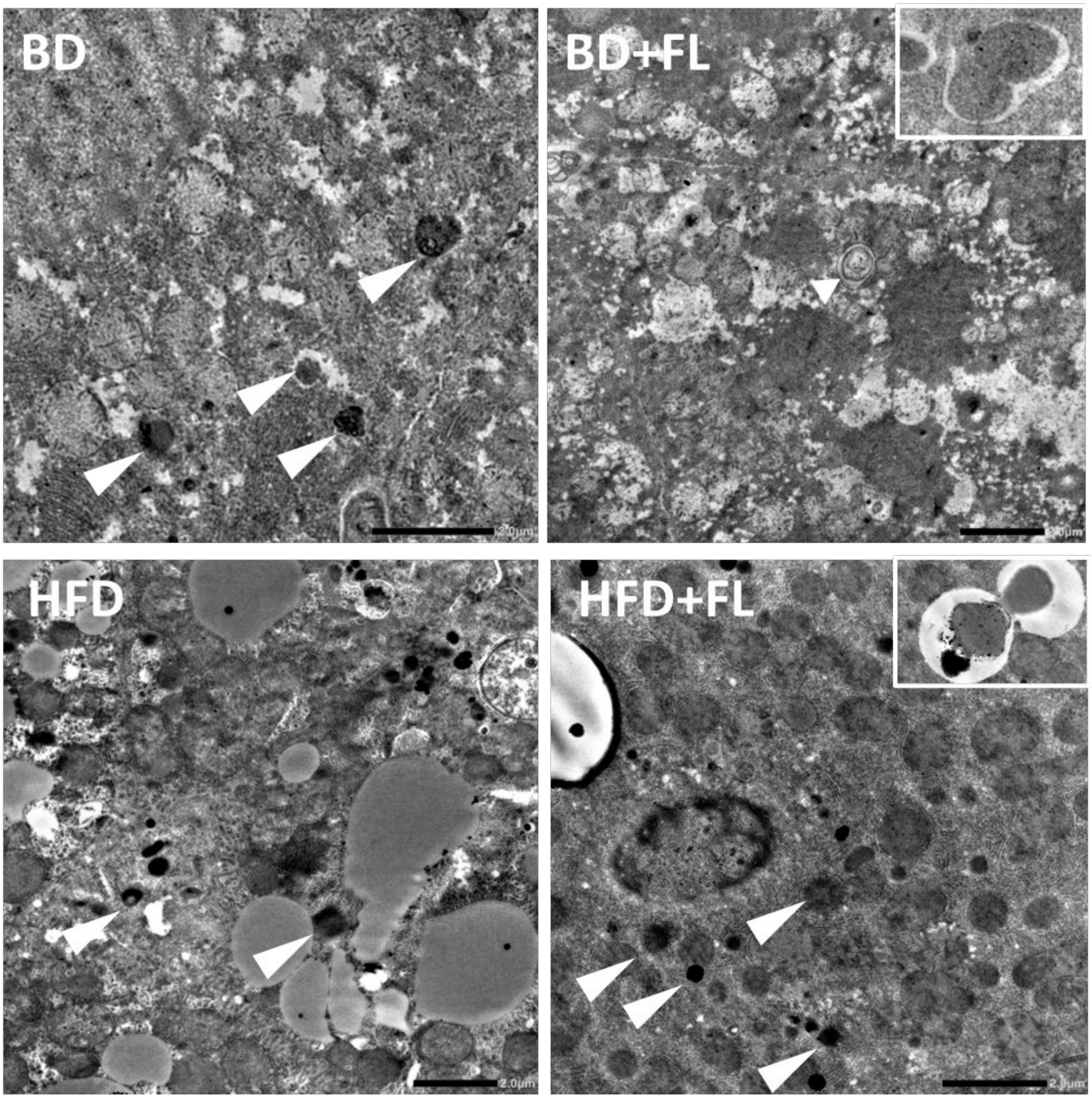

Figure S5. Representative TEM images of hepatocytes in rats. Autolysosomes (arrows) are scattered in the BD group. Autophagosomes, possibly mitophagy (short arrowhead; inset) are observed in the BF+FL groups. Variable sized-fatty droplets were observed in the HFD group. Variable-sized mitochondria and autolysosomes (arrowheads; inset) are noted in the HFD+FL groups. BD = basal diet, FL = flutamide, HFD = high fat diet. TEM, transmission electron microscopy. Bar=2.0 μm.

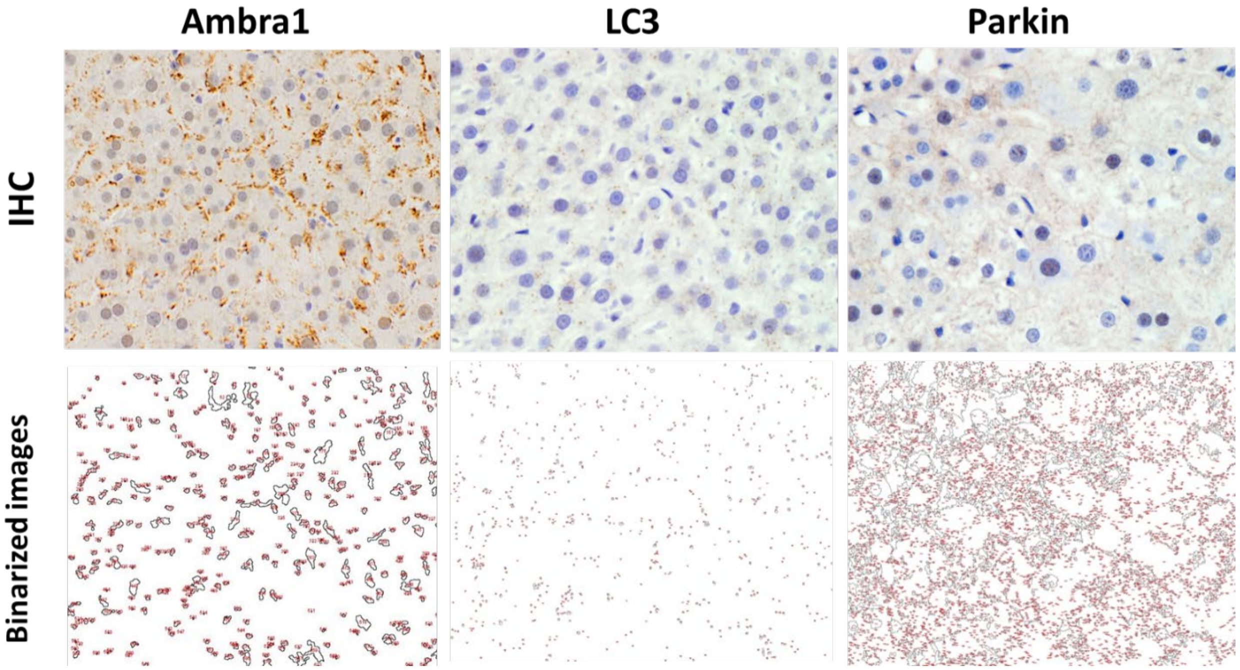

Figure S6. Detection of positive signals in immunohistochemistry in hepatocytes using Fiji (https://imagej.net/software/fiji/downloads). Representative images of immunostaining of AMBRA1, LC3, and Parkin in the upper panels. Fiji revealed the images of background (hematoxylin) stain and positive signals (DAB) of each target protein. Each of the DAB images was extracted for the target color using Colour Deconvolution. Finally, binarized positive signals are shown in the lower panels. Bar=50 µm. Abbreviations: AMBRA1 = autophagy and Beclin 1 regulator 1, DAB = 3,3'-diaminobenzidine, LC3 = microtubule-associated protein 1A/1B-light chain 3.
